# Profiling of Nutritionally Vital Bioactive Compounds in Emerging Green Leafy Vegetables: A Comparative Study

**DOI:** 10.3390/foods11233867

**Published:** 2022-11-30

**Authors:** Ramesh Kumar Saini, Min-Ho Song, Ji-Woo Yu, Jung-Hoon Lee, Hui-Yeon Ahn, Young-Soo Keum, Ji-Ho Lee

**Affiliations:** Department of Crop Science, Konkuk University, Seoul 143-701, Republic of Korea

**Keywords:** carotenoids, β-carotene, lutein, provitamin A, tocopherol, phytosterols, phytochemicals

## Abstract

Green leafy vegetables (GLVs), especially lettuce and spinach, are the key source of bioactive antioxidants in a diet. This research compared the contents and composition of lettuce and spinach bioactive compounds with emerging GLVs, moringa and fenugreek. Liquid chromatography (LC)-mass spectrometry (MS) with single ion monitoring (SIM) was used to examine carotenoids and tocols, while phytosterols were examined using gas chromatography (GC)-MS. Among the studied GLVs, the (all-*E*)-lutein was the most dominating carotenoid ranging between 31.3 (green/red lettuce)–45.3 % (fenugreek) of total carotenoids, followed by (all-*E*)-violaxanthin and (all-*E*)-β-carotene. Surprisingly, (all-*E*)-β-carotene, a provitamin A carotenoid, was the second most dominating carotenoid in moringa, accounting for 109.2 µg/g fresh weight (FW). Moreover, the significantly highest (*p* < 0.05; Tukey HSD) contents of total carotenoids (473.3 µg/g FW), α-tocopherol (83.7 µg/g FW), and total phytosterols (206.4 µg/g FW) were recorded in moringa. Therefore, moringa foliage may serve as an affordable source of nutritionally vital constituents in a diet.

## 1. Introduction

Clinical studies have shown the benefits of the consumption of fruits and vegetables in minimizing the occurrence of chronic diseases such as cardiovascular diseases (CVD), neurodegenerative diseases, type 2 diabetes (T2D), obesity, and cancer [1,2,3]. The bioactive antioxidants, including carotenoids and tocols obtained from fruits and vegetables, help prevent oxidative damage to cellular lipids, proteins, and DNA, thus minimizing the incidence of these chronic diseases [4,5,6,7].

Carotenoids are tetraterpenoid pigments universally produced by photoautotrophs. Provitamin A carotenoids (e.g., β-carotene) perform crucial roles in animals by serving as a dietary supply of provitamin A. Moreover, provitamin A and non-provitamin A carotenoids have antioxidant properties that protect against cancer, neurodegenerative diseases, metabolic syndromes (CVD and T2D), and photooxidative damage to the skin and eyes in animals [6,8].

As essential components of cellular lipids, tocopherols and tocotrienols (α-, β-, γ- and δ), also known as tocols, or vitamin E, scavenge free radicals and protect lipids from oxidative damage. Phytosterols are another group of health-promoting lipophilic compounds essential for maintaining blood levels of low-density lipoprotein (LDL) cholesterol and endothelial function [9]. The American Food and Drug Administration (FDA) states that in order to attain the health advantages of lowering blood total and LDL and lowering the risk of coronary heart disease, a dietary intake of 2 g/d of nonesterified plant sterols is required [10].

The risk of hunger and malnutrition is increasing at an alarming rate due to multiple factors, including low access to a healthy diet. High food prices due to low agricultural productivity in a changing climate and increasing populations cause a significant obstacle among low to medium- income consumers to balancing good nutrition with affordability. Therefore, there is a great need to find an affordable source of nutritionally vital constituents. Green leafy vegetables (GLVs), a component of a healthy diet, are key sources of nutritionally and nutraceutically vital constituents, including dietary fiber, minerals, vitamins, carotenoids, and polyphenolic compounds [11]. Among the commonly consumed culinary GLVs, lettuce (*Lactuca sativa* L.; Asteraceae family) and spinach (*Spinacia oleracea* L.; Amaranthaceae family) are the most widely consumed [12]. These GLVs are a rich source of carotenoids [13,14,15], polyphenols, and several other bioactive compounds [16,17].

Fenugreek (*Trigonella foenum graceum* L.; Fabaceae family) is an annual herb of medicinal importance [18]. Foliages are rich in carotenoids, ascorbate, and minerals [19,20]. Due to the unique aroma and flavor of seeds and dehydrated foliage, they are widely cultivated as a spice in the Middle East, Mediterranean countries, Russia, and Asia [21]. Moreover, in Asian countries, foliage is consumed raw, similarly to other GLVs [21].

Moringa (*Moringa oleifera* Lam.), also known as the “drumstick tree”, belonging to the Moringaceae family, is a tropical deciduous perennial tree cultivated in the tropical and subtropical regions of the world [22]. The immature pods, foliage, and flowers of this tree are used for culinary vegetables in tropical and subtropical countries. The moringa foliages are exceptionally rich in carotenoids [23], tocopherols [24], polyphenols [25], glucosinolates, and several other bioactives [26,27,28,29,30]. Moringa is emerging as a natural and affordable source of nutrients [31].

The carotenoid composition of GLVs, including lettuce, spinach, and moringa, have been widely investigated [14,15,32]. Moringa is considered a prominent source of carotenoids [23,27]; however, the carotenoid contents of moringa foliage were not widely compared with other commonly consumed GLVs. Comparative profiling may be helpful in the selection of nutrient-dense GLVs for culinary preparations. 

Moreover, only a few studies are available on the phytosterols profile of GLVs. In addition, previous studies have reported contrary observations regarding the phytosterol composition of spinach. For instance, Piironen et al. [33] recorded the dominance of spinasterol in spinach foliage. In contrast, Han et al. [34] reported the dominance of β-sitosterol in spinach and stigmasterol. Additionally, despite the vast consumption of fenugreek foliage, its bioactive composition has not been extensively investigated. Furthermore, the bioactive composition and antioxidant potential may be influenced by genetic, environmental, and geographical factors [35,36,37].

The carotenoid and tocopherol profile reported from GLVs is mainly based on HPLC-diode array (DAD) or ultraviolet (UV)-visible detection [14,21,32]. Mass spectrometry (MS) based quantification has several advantages over UV/Vis detection, including higher sensitivity and selectivity. Moreover, the interferences from co-eluting compounds can be avoided in an MS-based analysis [38].

Considering the above facts, in the present study, the contents and composition of major lipophilic compounds, including carotenoids, tocols, and phytosterols of lettuce and spinach, were compared with the emerging GLVs moringa and fenugreek. For this comparison, three carotenoid-rich green (*cv*. Esse), green/red (*cv*. Jeokcima), and red (*cv*. Super caesar red) lettuce cultivars were particularly selected based on our previous report [14]. Liquid chromatography (LC)- MS with single ion monitoring (SIM) was used to examine carotenoids and tocols, while phytosterols were analyzed using gas chromatography (GC)-MS. The profiling of nutritionally vital bioactive compounds of commonly consumed GLVs can significantly contribute to identifying nutritionally enriched vegetables for food formulations and dietary recommendations.

## 2. Materials and Methods

### 2.1. Plant Material, Reagents, and Standards

Seeds of green (*cv*. Esse), green/red (*cv*. Jeokcima), and red (*cv*. Super caesar red) lettuce (*Lactuca sativa* L.) cultivars, and *Moringa oleifera* Lam. were obtained from the Asia Seeds Co., Ltd., Seoul, Korea. The spinach (*Spinacia oleracea* L.) and fenugreek (*Trigonella foenum-graecum* L.) seeds were obtained from a local supplier. The cool season vegetables, lettuce, spinach, and fenugreek were grown between April and May 2022 (average high temperature of 10–20 °C), while the tropical plant, moringa, was grown between June and August 2022 (mean temperature of 25–30 °C) in an open space at an ambient temperature and humidity. Seeds of lettuce, spinach, and fenugreek were shown in three replicates in ports measuring 28 × 48 cm, filled with a commercial potting mix (Asia Seeds Co., Ltd. Seoul, Korea), comprising 300–500 ppm NPK and 30–100 cmol/kg of cation exchange capacity (CEC).

After the germination of lettuce, fenugreek, and spinach (a week after sowing), 10–18 plants were maintained in each pot. In the case of moringa, only two plants were maintained in each pot of 28 × 48 cm (five replicates). The plants were raised without applying any fertilizers and pesticides. Lettuce, spinach, and fenugreek foliage was harvested seven weeks after showing, while moringa foliage was harvested 12 weeks after showing. Healthy foliages from the entire plant were collected, cleaned, and stored in a −80 °C deep freezer, until analysis.

Authentic standards of (all-*E*)-β-carotene, 5-β-cholestan-3α-ol (internal standard), campesterol (24α-methyl cholesterol), β-sitosterol (24α-ethyl cholesterol), stigmasterol, and α-spinasterol were obtained from Merck Ltd., Seoul, South Korea.

A tocols mix (δ-, γ-, β-, and α-tocopherol and tocotrienol) solution was purchased from ChromaDex, Inc., Irvine, CA, USA. (all-E)-zeaxanthin used in this study was purified from corn seeds, while (all-*E*)-violaxanthin, 9-*Z*-neoxanthin, (all-*E*)-lactucaxanthin, and (all-*E*)-lutein were purified from lettuce using our established protocol [39]. (all-E)-luteoxanthin was prepared from (all-*E*)-violaxanthin using an acid-catalyzed reaction [40].

The organic solvents used for extraction and analysis were of LC grade and procured from J.T. Baker^®^, Suwon-Si, Korea.

### 2.2. Extraction of Crude Lipids (Lipophilic Compounds)

The lipophilic bioactives, including carotenoids, tocols, and phytosterols, were concurrently extracted from fresh foliage using our optimized method [14] with minor modifications. The extraction procedure is outlined in Figure S1. To minimize the oxidative degradation of lipophilic compounds, synthetic antioxidant butylated hydroxytoluene (BHT) was added to the extraction solvent (0.1% *w*/*v*) [41]. Tocols and carotenoids were analyzed without hydrolysis, as hydrolysis can degrade these compounds [42].

An 0.7 mL aliquot of the extracted sample was hydrolyzed and converted to trimethylsiloxy [−O-Si(CH_3_)_3_; TMS] derivatives (Figure S2) [42], and utilized for GC-MS analysis of phytosterols.

### 2.3. LC-SIM-MS Analysis of Tocols and Carotenoids

The LC-SIM-based MS approach using a LCMS-9030 quadrupole time-of-flight (Q-TOF) mass spectrometer was used to analyze the carotenoids and tocols (Shimadzu, Tokyo, Japan). The LC-MS parameters are outlined in Table 1. Additionally, the selected ion monitoring (SIM) parameters are shown in Table 2.

### 2.4. GC-MS Analysis of Phytosterols

Phytosterols were analyzed using QP2010 SE GC-MS (Shimadzu, Tokyo, Japan) after silylation with trimethylsiloxy groups [−O-Si(CH_3_)_3_; TMS] group. The analytical details are summarized in Table 3. The mass fragmentation pattern of obtained phytosterols was compared with authentic standards and reference databases, including Wiley9, NIST08, and NIST08S.

### 2.5. Statistical Analysis and Quality Control

A total of three separate replicate extractions and analysis were performed for each GLV. IBM SPSS statistics (version 25) software was utilized to perform the one-way analysis of variance (ANOVA), considering a significance level of 0.05 (Tukey HSD).

The LC-SIM-MS and GC-MS method used for the quantification of carotenoids and tocols, and phytosterols, respectively, was validated in terms of accuracy, linearity, and precision [44,45]. The limits of detection (LOD) and limits of quantitation (LOQ) were determined according to the following equations [44].

(1) $$LOD=\frac{3.3\;\times \;Standard\;deviation\;of\;the\;response}{Slope\;of\;the\;calibration\;curve\;}$$

(2) $$LOQ=\frac{10\;\times \;Standard\;deviation\;of\;the\;response}{Slope\;of\;the\;calibration\;curve}$$

The instrumental precision (intra-day and inter-day) for retention time and peak area count was calculated from the data obtained with numerous injections of the same concentration (within the working range) and expressed as the percentage of the coefficient of variation (% CV). Six replicate injections of the same concentration in a single day were performed to measure the intra-day precision. In contrast, standards were injected six times over the course of two separate, non-consecutive days to establish the inter-day precision.

## 3. Results and Discussion

### 3.1. Method Validation

The LC-SIM-MS and GC-MS methods used for the quantification of carotenoids and tocols, and phytosterols, respectively, were validated in terms of accuracy, linearity, and precision [44,45]. The coefficient of variation (CV), also known as relative standard deviation (RSD), was recorded below 9.25 and 0.32% (intra-day and inter-day) for the peak areas count and retention times, respectively, for carotenoids and tocopherol using LC-SIM-MS (Table S1). For the bioanalytical method, a CV below 15% is acceptable [46]. The calibration curves also showed a high coefficient of correlation (r^2^; >0.999–1.000) between standard concentrations and peak area counts.

Similarly, the CV values were recorded below 4.78 and 0.07% (intra-day and inter-day) for peak area count and retention times, respectively, for sterols using GC-MS (Table S2). The calibration curves also showed a high coefficient of correlation (r^2^; >0.991–1.000) between standard concentrations and peak area counts. These observations supported the good accuracy, linearity, and precision of the deployed LC-SIM and GC-MS methods.

### 3.2. Carotenoids Composition

**Figure 1:**
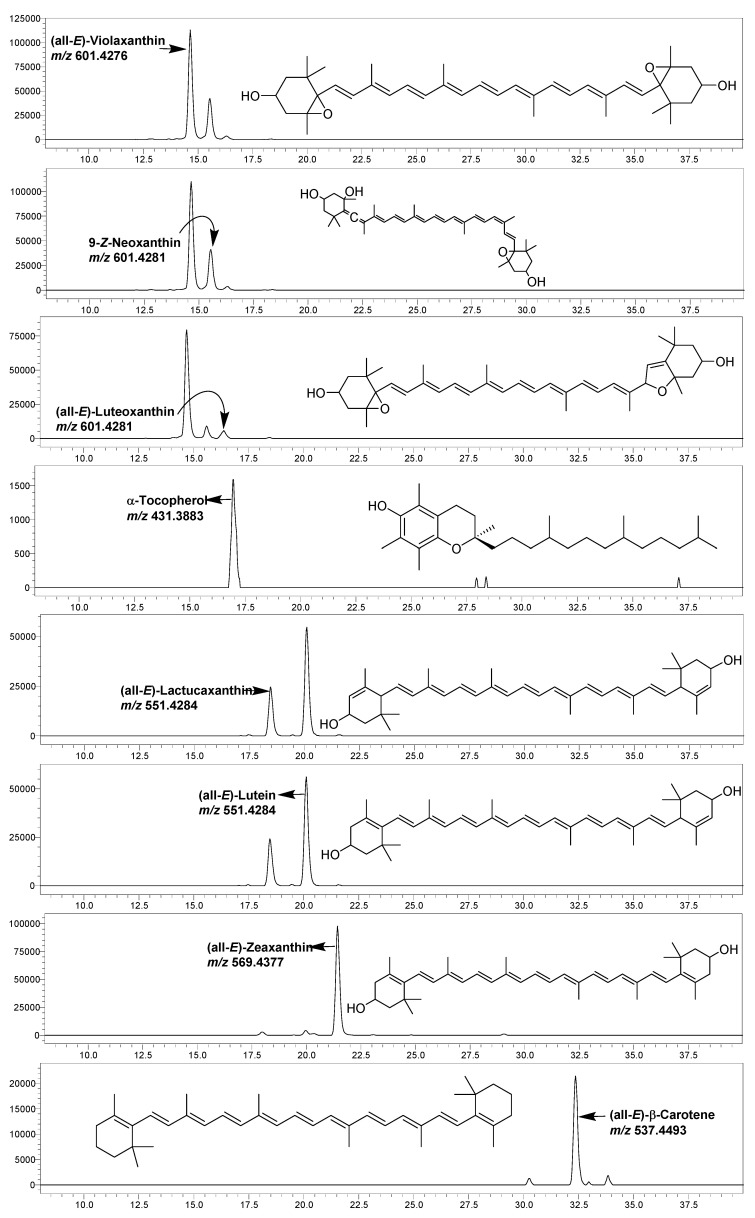
The representative LC-SIM-MS chromatograms of various carotenoids and α-tocopherol identified in studied GLVs.

Among the lipophylic vitamins and other bioactive compounds in GLVs, carotenoids are the most vital constituents that substantially impact the nutritional value and attractive color of food [47]. In this study, seven major carotenoids, viz., (all-*E*)-violaxanthin (retention time (RT) of 14.63 min), 9-Z-neoxanthin (15.54 min), (all-*E*)-luteoxanthin (16.30 min), (all-*E*)-lactucaxanthin (18.41 min), (all-*E*)-lutein (19.96 min), (all-*E*)-zeaxanthin (21.43 min), and (all-E)-β-carotene (32.36 min) were identified and quantified using the LC-SIM-based MS method (Figure 1, Table 4).

Among the studied foliages, the (all-*E*)-lutein was the most dominating carotenoid, ranging between 31.3 (green/red lettuce)–45.3 % (fenugreek) of total carotenoids, followed by (all-*E*)-violaxanthin and (all-*E*)-β-carotene. Surprisingly, (all-*E*)-β-carotene was the second most dominating carotenoid in moringa, accounting for 109.2 µg/g FW.

Provitamin A carotenoids, especially β-carotene, are nutritionally vital for their vitamin A activity [47], while non-provitamin A carotenoids (e.g., xanthophylls) are important for their antioxidative activities, which prevent chronic and metabolic disorders [6,8]. Considering the substantially high amount of (all-*E*)-β-carotene in the moringa foliage, its consumption can help improve the body’s vitamin A status. The recommended dietary allowance (RDA) of vitamin A is 900 retinol equivalents (RE; 900 RE = 900 μg of retinol or 10,800 µg of dietary β-carotene) for adult men [48]. Considering the (all-*E*)-β-carotene contents of 109.2 µg/g FW (9.1 μg of RE/g FW), consumption of 100g fresh moringa foliage can supply 101.1% of the RDA of vitamin A.

In addition to the (all-*E*)-β-carotene, significantly high contents of (all-*E*)-lutein (204.6 μg/g FW) and (all-*E*)-zeaxanthin (84.2 μg/g FW) were recorded in moringa, compared to other studied GLVs. Lutein and zeaxanthin are macular pigments acting as blue light filters protecting the retina and, therefore, vision [49]. Studies have shown that increased lutein and zeaxanthin intake can help maintain ocular health [49]. Thus, among the GLVs researched in the present study, moringa is the most vital to increase the intake of lutein and zeaxanthin in a diet.

Moringa is a fast-growing, drought-resistant, and easy-to-cultivate tropical plant. Moringa foliage is an affordable source of nutrients for the populations of developing countries where malnutrition persists [50]. Moreover, moringa foliage can be harvested during the dry season when no other GLVs are readily available [51]. In the present study, the total carotenoid contents (TCC) of moringa (473.3 µg/g FW) was recorded nearly four times higher than lettuce (86.3–116.1 µg/g FW), and two times higher than spinach (218.5 µg/g FW) and fenugreek (214.2 µg/g FW). Suggesting that moringa is a more significant source of carotenoids than other commonly consumed GLVs.

We have previously investigated the carotenoid profile of 23 diverse lettuce cultivars [14]; three carotenoid-rich cultivars with green, green/red, and red foliage were selected in the present investigation to compare their profile with other commonly consumed GLVs. The carotenoid profile and contents recorded in the present investigation are in agreement with our previous report [14]. Similarly, the carotenoid profile of lettuce cultivars recorded in the present study agrees with our previous report from minimally processed ready-to-eat baby-leaf vegetables, including green and red Romaine lettuce [13]. Moreover, we have previously analyzed the carotenoid profile of eight diverse moringa cultivars grown in India [52]. In that study, 118.6 (*cv*. PAVM-1)–231.5 µg/g FW (*cv*. Bhagya) of (all-*E*)-β-carotene contents was recorded, which agrees with the present study.

The *all trans* (all-*E*) is the major form of β-carotene in green leafy vegetables, including spinach [15]. In a previous study, (all-*E*) form accounted for 86.3–87.6 % of β-carotene in spinach with a minor presence of 9-*Z*- and 13-*Z*-β-carotene [15]. In that study, 36.2, 56.6, 38.3, and 29.2 µg/g FW of (all-*E*)-β-carotene was recorded from authentic field-grown, greenhouse-grown, fresh commercial, and frozen commercial spinach, respectively [15]. These results are in agreement with the (all-*E*)-β-carotene recorded from spinach in the present study.

A previous study on fenugreek foliage reported the presence of a significant amount of β-cryptoxanthin (75 µg/g FW), zeaxanthin (82 µg/g FW), and γ-carotene (41 µg/g FW), with the TCC of 712 µg/g FW [21]. In contrast, we recorded 5.16 µg/g FW of (all-*E*)-zeaxanthin, with the TCC of 214.2 µg/g FW, while β-cryptoxanthin and γ-carotene were not detected. Lutein and zeaxanthin are positional isomers. In general, in most green leafy vegetables, including fenugreek, lutein dominates with a minor presence of zeaxanthin [32].

Recently, Lee et al. [38] analyzed the content of carotenoids and tocopherols in 26 green leafy vegetables, including spinach, moringa, and fenugreek. The β-carotene contents reported in that study from spinach (43 µg/g FW) and moringa (108 µg/g FW) foliage are in agreement with the present study. However, we recorded a substantially low amount of violaxanthin and neoxanthin compared to that study.

A significant variation in carotenoid content obtained in the present study from previous reports is probably driven by the harvesting stage, as well as cultivational, environmental, and genetic factors, which influence the carotenoid contents and crop compositions [35].

### 3.3. Tocols Composition

Tocols includes four naturally occurring tocopherols (δ-, γ-, β-, and α-) and four tocotrienols (δ-, γ-, β-, and α-) [53]. In this study, the LC-SIM-MS-based method was employed for analyzing the tocol contents and composition. Among the studied foliages, 22.0 (spinach)–87.7 µg/g FW (moringa) of α-tocopherol was recorded (Table 4), while other forms were not detected in a substantial amount.

The RDA for both men and women is 15 mg/day of α-tocopherol [54]. Among the naturally occurring tocols, α-tocopherol is considered the highest 100% vitamin E activity (1 mg α-tocopherol = 1 α-TE), followed by β-tocopherol (50%), α-tocotrienol (30%), γ-tocopherol (10%), β-tocotrienol (5%), and δ-tocopherol (3%) [54]. Vegetable oil, especially wheat gem oil, is the major source of vitamin E in the body [54]. However, considering the highest α-tocopherol contents of 87.3 µg/g FW, which is 2–4 times higher than other studied GLVs, 100g of fresh moringa foliage can supply 55.8% of the RDA of vitamin E. Moreover, α-tocopherol is well known for its chain-breaking antioxidant functions that prevent the propagation of lipid peroxidation, thus minimizing oxidative stress-related diseases [54,55,56,57].

The previous reports of α-tocopherol contents from GLVs have shown substantial variation. In a study by the US Department of Agriculture (USDA), 2.2–5.5 and 19.6 µg/FW of α-tocopherol was recorded from various types of lettuce, and spinach, respectively [58]. We also recorded 22.0 µg/FW of α-tocopherol from spinach, while α-tocopherol contents from studied lettuce are substantially higher (25.1–30.1 µg/FW) in the present study. The contents of α-tocopherol significantly depend on the leaf position in the head of matured lettuce. For instance, a significantly higher amount of α-tocopherol (16.2 µg/g FW) was recorded from the outer leaf, compared to the middle (5.1 µg/g FW) and central leaves (8.5 µg/g FW) of the lettuce head [59]. We harvested lettuce and other studied GLVs at the baby-leaf stage (7 weeks after showing). At this stage, all the leaves received a nearly equal amount of sunlight; this is the probable reason for the high amount of α-tocopherol recorded in the present investigation.

Recently, Lee et al. [38] recorded 183 µg/g FW of α-tocopherol in moringa foliage. Similarly, in our previous study, 173 µg/g FW of α-tocopherol was recorded from moringa (cv. PKM-1) grown in the field in India, which is higher than the contents recorded in the present study (83.7 µg/g FW). However, α-tocopherol contents recorded from moringa in the present study are in good agreement with the report of Ching and Mohamed [60], who recorded 90 µg/g FW α-tocopherol in moringa foliage.

A recent study on fenugreek foliage recorded 31.2 µg/g FW of α-tocopherol [38], which is lower than documented in the present study (43.6 µg/g FW).

### 3.4. Phytosterol Composition

**Figure 2:**
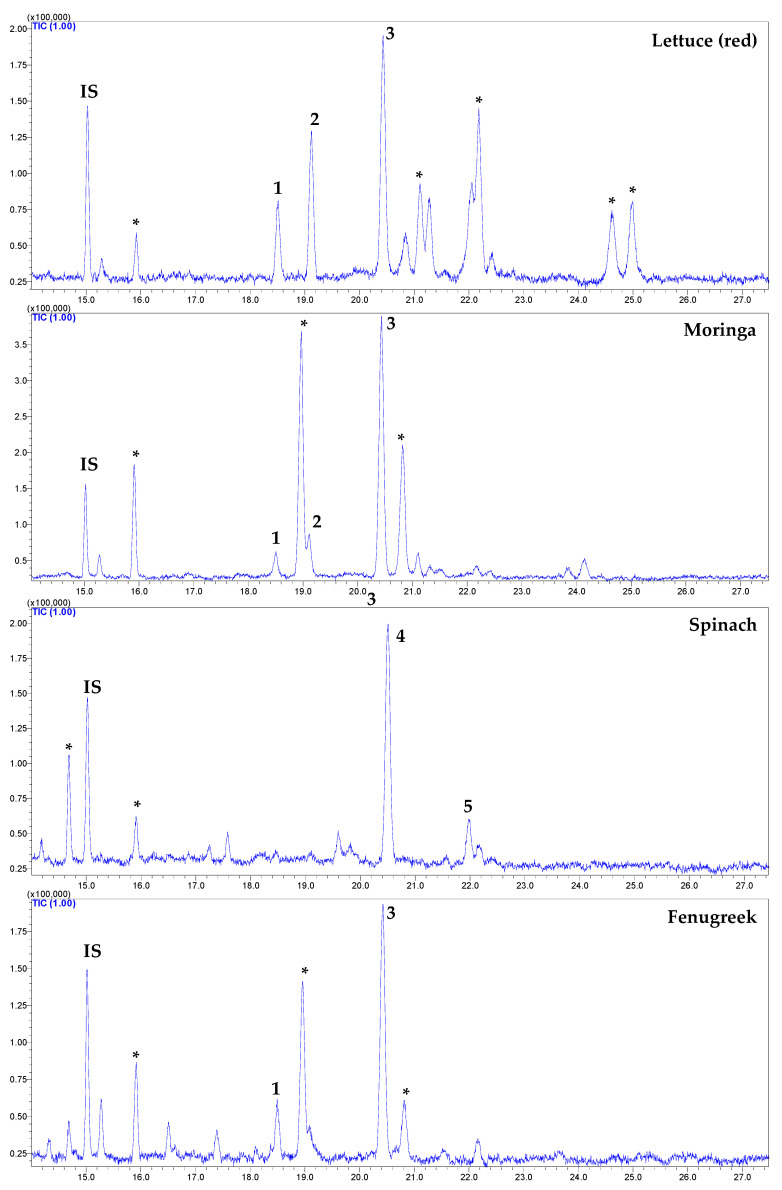
The characteristic GC-MS-total ion chromatograms of phytosterols identified in studied GLVs. IS: internal standard. 1, campesterol; 2, stigmasterol; 3, β-sitosterol; 4, α-spinasterol; and 5, 22, 23-dihydrospinasterol. * not a sterol compound.

In this study, lipophilic extract derivatized with trimethylsiloxy groups [−O-Si(CH_3_)_3_; TMS] and analyzed utilizing GC-MS revealed the presence of campesterol (RT-18.494), stigmasterol (RT-19.017), β-sitosterol (RT-20.419), spinasterol (RT-20497), and 22,23-dihydrospinasterol (RT-21.966) in the studied samples (Figure 2).

The trimethylsilanol (TMS-OH) loss, yielding a major ion fragment at *m*/*z* [M-90]^•+^, is a characteristic often utilized for the mass spectrometric identification of sterols [61]. Additionally, the Δ5-steryl-TMS, such as β-sitosterol, stigmasterol, and campesterol, give a distinctive fragmentation involving loss of TMS-OH with the C-3, C-2, and C-1 of the sterol A-ring, resulting in dominant ions at *m*/*z* 129 and *m*/*z* [M-129]^•+^ for the remaining part of the sterol compound (Figures S3 and S4) [61]. In contrast, the Δ7-steryl TMS, such as α-spinasterol and 22-23 dihydrospinasterol, produce distinctive ion [M-141]^+^ by losing the side chain with two hydrogens (Figure S4) [62].

In this study, β-sitosterol was the most dominating phytosterol among the studied GLVs (except spinach), ranging between 53.3 (green lettuce)−85.7 (fenugreek) µg/g FW, which accounted for 46.0 (green/red lettuce)−85.4% (fenugreek) of total phytosterol (Table 5). Interestingly, α-spinasterol was the most dominating sterol in spinach, accounting for 81.9% of total phytosterols, while other commonly occurring forms were not detected.

In the present study, the significantly highest (*p* < 0.05; Tukey HSD) contents of total phytosterols were recorded from moringa foliage (206.4 µg/g FW), followed by green/red lettuce (153.4 µg/g FW), and red lettuce (135.7 µg/g FW) (Table 5). These results show that moringa foliages are a rich source of phytosterols compared to other commonly consumed GLVs.

In agreement with the present study, β-sitosterol is reported as the most dominating phytosterol in fruits and vegetables, except for spinach and a few others [33]. In spinach, 102 µg/g FW of total phytosterols were reported by Piironen et al. [33], with the dominance of spinasterol (62%) and dihydrospinasterol (27%). Interestingly, we also recorded 109.9 µg/g FW of total phytosterols in spinach with the α-spinasterol and 22,23-dihydrospinasterol. In contrast, Han et al. [34] reported the dominance of β-sitosterol in spinach (54 µg FW) and stigmasterol (29 54 µg FW).

Limited studies are available on the phytosterol composition of green leafy vegetables. We have previously recorded the dominance of β-sitosterol in herbs such as *Kaempferia parviflora* Wall. Ex Baker (30.6–36.6 µg/g FW) [63] and perilla (*Perilla frutescens* Britt.; 27.7–37.9 µg/g FW) [64], with campesterol as a minor phytosterol.

## 4. Conclusions

High food prices cause a significant obstacle among low- to medium-income consumers to balancing good nutrition with affordability. Thus, there is a great need to find an affordable source of nutritionally vital constituents. This research compared the contents and composition of lettuce and spinach bioactive compounds with the emerging GLVs moringa and fenugreek. Among the three cultivars of lettuce foliage studied, moringa, spinach, and fenugreek, the significantly highest (*p* < 0.05; Tukey HSD) contents of total carotenoids (473.3 µg/g FW), α-tocopherol (83.7 µg/g FW), and total phytosterols (206.4 µg/g FW) were recorded in moringa. Considering the (all-*E*)-β-carotene (a provitamin A carotenoid) contents of 109.2 µg/g FW, consumption of 100g fresh moringa foliage can supply 101.1% of the RDA of vitamin A. These observations suggest that moringa is a more significant source of carotenoids, α-tocopheol, and phytosterols than other commonly consumed GLVs studied in the present investigation.

In this study, β-sitosterol was the most dominating phytosterol among the studied GLVs (except for spinach). Interestingly, α-spinasterol was the most dominating sterol in spinach, accounting for 81.9% of total phytosterols. Limited studies are available on the phytosterol composition of GLVs. In the future, more GLVs will need to be screened for their phytosterol composition.

## Figures and Tables

**Table 1:** LC-SIM-MS parameters used for the analysis of carotenoids and tocols.

**HPLC System: Nexera 40 Series UHPLC (Shimadzu, Tokyo, Japan)**	
Column	YMC C30 carotenoid column (150 mm × 4.6 mm, 3 μm; YMC, Wilmington, NC, USA), maintained at 20 °C
Mobile phase ^1^	A- methanol/water (95/5; *v/v*) with 5 mM ammonium formate B- methyl tertiary butyl ether (MTBE)/methanol/water (90/7/3, *v/v/v*), with 5 mM ammonium formate
Gradient program ^1^	0 % B (0 min) –100 % B (45 min); 5-min post-run (0 % B)
Flow rate	0.5 mL/min
Injection volume	2 µL
**MS system: LCMS-9030 quadrupole time-of-flight (q-TOF) mass spectrometer (Shimadzu, Tokyo, Japan)**	
Nebulizing gas flow	3 L/min
Drying gas flow	10 L/min
Interface temperature	400 °C
Corona needle voltage	4.0 kv
Heat block Temperature	300 °C
DL temperature	300 °C
Ionization	Atmospheric-pressure chemical ionization (APCI; Positive mode)
MS program	min- diverter valve to drain 8.0 min- diverter valve to MS
Q1 resolution	±20 ppm
Screening mode	Single ion monitoring (SIM)
Data acquisition (sampling)	1.85625 Hz

^1^ used with minor modifications as suggested by the column supplier (YMC, Wilmington, NC, USA) to separate the carotenes and xanthophylls; The MS parameters were optimized to obtain the highest response.

**Table 2:** The transition (*m*/*z*) utilized for selected ion monitoring (SIM) of carotenoids and tocols.

Compound Class	Compound	Transition (*m*/*z*) *
Carotenoids	(all-*E*)-violaxanthin	601.4276
	9-*Z*-neoxanthin	601.4281
	(all-*E*)-luteoxanthin	601.4281
	(all-*E*)-lactucaxanthin	551.4284
	(all-*E*)-lutein	551.4284
	(all-*E*)-zeaxanthin	569.4377
	(all-*E*)-β-carotene	537.4493
Tocols	δ-tocopherol	402.3488
	δ-tocotrienol	397.3113
	γ- tocopherol	416.3669
	γ- tocotrienol	411.3268
	δ-tocopherol	416.3669
	δ-tocotrienol	411.3268
	α- tocopherol	431.3883
	α- tocotrienol	425.3423

* Selected based on the most prominent protonated precursor ion obtained from the standard compounds.

**Table 3:** GC-MS conditions used for the analysis of phytosterols.

**Gas Chromatograph**			
Injection temperature	260 °C		
Column over temperature	150 °C		
Carrier gas	Helium		
Injection mode	split		
Flow control mode	Liner velocity		
Total flow	8.6 mL/min		
Pressure	86.5 kPa		
Column flow	0.93 mL/min		
Liner velocity	36.7 cm/s		
Purge flow	3.0 mL/min		
Column over temperature	Rate (°C/min)	Final temperature (°C)	Hold time (min)
	-	150	1
	20	300	30
Column	DB-5ms (30 m, 0.25 μm, 0.25 mm ID; Agilent Technologies Canada, Inc.		
Total program time	38.5 min		
**Mass spectrometer**			
Interface temperature	280 °C		
Ion source temperature	260 °C		
Solvent cut time	3 min		
Start time and end time	6 min and 38 min		
Acquiring mode	Scan		
Event time	30 s		
Scan speed	2500		
Start and end *m*/*z*	50.00 and 650.00		

The GC-MS conditions were adopted from our previous studies [43], with minor modifications.

**Table 4:** The contents (µg/g FW) of carotenoids and α-tocopherol in studied GLVs.

Compounds	RT (min)	Lettuce (Green)	Lettuce (Green/Red)	Lettuce (Red)	Moringa	Spinach	Fenugreek
(all-*E*)-violaxanthin	14.63	27.0 ± 0.84 ^d^	24.3 ± 1.40 ^d^	25.9 ± 1.27 ^d^	37.0 ± 0.72 ^c^	57.1 ± 1.49 ^a^	46.2 ± 4.32 ^b^
9-*Z*-neoxanthin	15.54	6.73 ± 0.55 ^e^	6.23 ± 0.47 ^e^	9.23 ± 0.83 ^d^	35.8 ± 0.39 ^a^	23.2 ± 0.10 ^b^	17.9 ± 0.81 ^c^
(all-*E*)-luteoxanthin *	16.30	3.65 ± 0.65 ^b^	3.88 ± 0.23 ^b^	3.62 ± 0.41 ^b^	2.57 ± 0.33 ^b^	0.28 ± 0.05 ^c^	11.3 ± 1.97 ^a^
(all-*E*)-lactucaxanthin	18.41	9.52 ± 0.92 ^b^	8.66 ± 0.24 ^b^	17.1 ± 1.05 ^a^	n.d.	n.d.	n.d.
(all-*E*)-lutein	19.96	29.5 ± 2.21 ^e^	27.0 ± 1.93 ^e^	37.0 ± 1.50 ^d^	204.6 ± 1.86 ^a^	89.9 ± 0.35 ^c^	97.1 ± 4.48 ^b^
(all-*E*)-zeaxanthin	21.43	3.24 ± 0.24 ^c^	2.74 ± 0.32 ^cd^	2.46 ± 0.24 ^cd^	84.2 ± 1.73 ^a^	1.47 ± 0.05 ^d^	5.16 ± 0.17 ^b^
(all-*E*)-β-carotene	32.36	14.2 ± 0.31 ^e^	13.5 ± 0.66 ^e^	20.8 ± 1.38 ^d^	109.2 ± 5.09 ^a^	46.6 ± 3.28 ^b^	36.6 ± 1.51 ^c^
Total carotenoids		93.7 ± 5.72 ^d^	86.3 ± 5.25 ^d^	116.1 ± 5.85 ^c^	473.3 ± 5.88 ^a^	218.9 ± 1.34 ^b^	214.2 ± 9.32 ^b^
α-tocopherol	16.92	30.1 ± 3.31 ^c^	25.1 ± 0.85 ^d^	25.18 ± 2.75 ^d^	83.7 ± 0.78 ^a^	22.0 ± 1.56 ^d^	43.6 ± 2.61 ^b^

Values are the mean ± standard deviation (SD) of four replicate analyses. The various superscript letters (i.e., a, b, c, d, and e) show statistically significant differences (*p* < 0.05, Tukey HSD) among the different GLVs. RT: Retention time. * calculated contents are (all-*E*)-violaxanthin equivalent.

**Table 5:** The phytosterol contents (µg/g FW) in studied GLVs.

Phytosterols	RT (min)	Lettuce (Green)	Lettuce (Green/Red)	Lettuce (Red)	Moringa	Spinach	Fenugreek
Campesterol	18.494	11.9 ± 1.09 ^cd^	17.2 ± 1.35 ^b^	21.4 ± 1.22 ^a^	10.0 ± 3.15 ^d^	n.d.	14.6 ± 0.79 ^bc^
Stigmasterol	19.107	40.9 ± 6.43 ^b^	65.6 ± 7.12 ^a^	40.6 ± 1.23 ^b^	20.4 ± 0.63 ^c^	n.d.	n.d.
β-Sitosterol	20.419	53.3 ± 5.57 ^d^	70.6 ± 7.76 ^c^	73.7 ± 5.72 ^cd^	175.9 ± 6.22 ^a^	n.d.	85.7 ± 4.63 ^b^
α-Spinasterol	20.497	n.d.	n.d.	n.d.	n.d.	83.4 ± 6.05 ^a^	n.d.
22, 23-Dihydrospinasterol *	21.966	n.d.	n.d.	n.d.	n.d.	18.5 ± 2.13 ^a^	n.d.
Total Phytosterol		106.1 ± 12.0 ^c^	153.4 ± 15.8 ^b^	135.7 ± 6.24 ^b^	206.4 ± 8.27 ^a^	101.9 ± 5.48 ^c^	100.4 ± 3.85 ^c^

Values are the mean ± standard deviation (SD) of four replicate analyses. The various superscript letters (i.e., a, b, c, and d) show statistically significant differences (*p* < 0.05, Tukey HSD) among the different GLVs. * identified based on reference databases, and calculated contents are α-Spinasterol equivalent.

## Data Availability

Data is contained within the article.

## Supplementary Materials

The following are available online at https://www.mdpi.com/article/10.3390/foods11233867/s1, Figure S1: Outline of the Method Used for Extraction of Crude Lipids, Figure S2: Outline of Methods Used for the Hydrolysis and Silylation of Sterols for GC-MS, Figure S3: A characteristic mass fragmentation pattern of β-Sitosterol (trimethylsiloxy (TMS) derivative) observed in the present investigation, Figure S4: The characteristic GC-MS-fragmentation pattern of phytosterols identified in studied GLVs, Table S1: Method analytical and validation parameters for LC-SIM-MS analysis of carotenoids and tocopherols, Table S2: Method analytical and validation parameters for GC-MS analysis of sterols.
