# Supplementary material for: Profiling of Nutritionally Vital Bioactive Compounds in Emerging Green Leafy Vegetables: A Comparative Study
Source: Foods. 2022 Nov 30;11(23):3867. doi: 10.3390/foods11233867 (PMC9736515; doi:10.3390/foods11233867)

Figure S1. Outline of the Method Used for Extraction of Crude Lipids

2g fresh sample in 50 ml falcon tube

↓

Samples were homogenized with 25 ml (acetone/ethanol/cyclohexane, 1:1:2, v/v) containing 0.1 % butylated hydroxytoluene (BHT: w/v; antioxidant),

↓

Bath sonication (JAC-2010; 300 w, 60 Hz, for 10 min) followed by ultra-shaking for 2 min in collomix viba x.30 (Tinting Solutions B.V., Nederland) for the efficient disintegration and complete extraction

↓

Vacuum filtered, and pellets were extracted again with 20 ml extraction solvent

↓

Filtrate containing lipophilic compounds were pooled, transferred to a 250 ml round bottom flask, and vacuum-dried in a rotary evaporator at 35 °C

↓

Lipophilic compounds were recovered in 4 ml acetone containing 0.1% BHT and transferred to 5 ml glass vial fitted with a Teflon-lined screw cap. These crude lipids were utilized as follows:

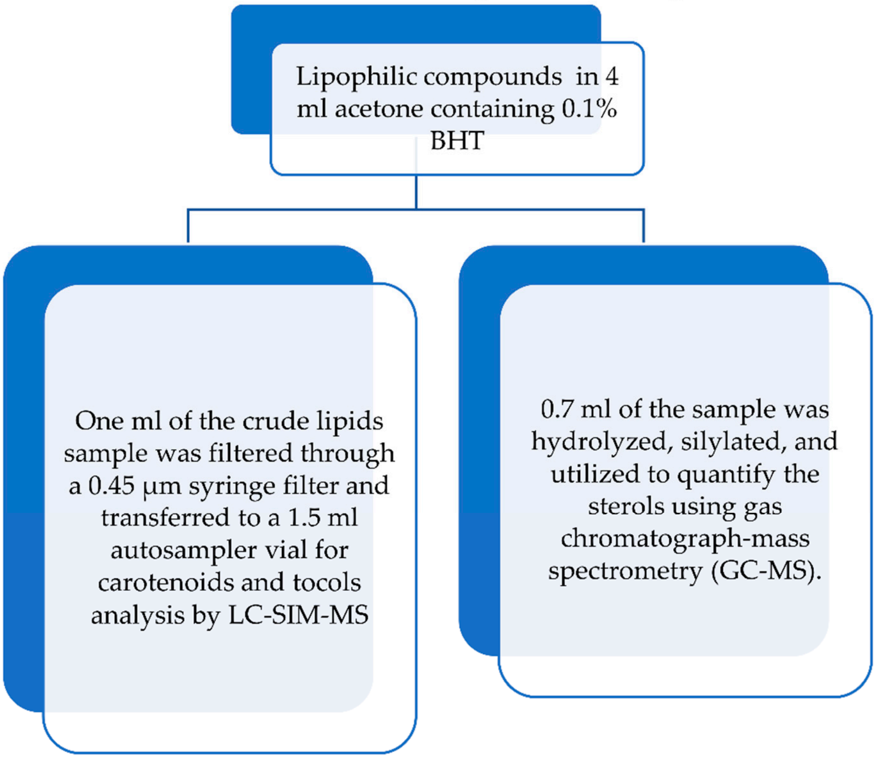

Figure S2. Outline of Methods Used for the Hydrolysis and Silylation of Sterols for GC-MS

0.7 ml of crude lipids sample was transferred into a 5 ml glass vial fitted with a Teflon-lined screw cap (10 μg of internal standard for phytosterols, 5β-cholestan-3α-ol was added)

↓

Contents were evaporated to dryness using a rotary evaporator at 35 °C

↓

2 ml of 0.5 M methanolic KOH were added and placed in a water bath at 85 °C for 15 min (for hydrolysis)

↓

Hydrolyzed samples were immediately cooled in ice, and partitioned with 2 ml of cyclohexane and 1 ml of 1 M NaCl

↓

1 ml from the upper cyclohexane phase containing phytosterols was carefully transferred into a new 5 ml Teflon lined glass tube and vacuum-dried in a rotary evaporator at 35 °C

↓

For silylation of phytosterols, 1 ml pyridine, and 50 μl of N,O-bis(trimethylsilyl)trifluoroacetamide (BSTFA) containing 1% trimethylchlorosilane (TMCS) and added and incubated at 60 °C for 60 min (generally use 45 min)

↓

After incubation, contents were cooled in ice, filtered through a 0.45 μm PTFE syringe filtered and transferred to a 1.5 ml autosampler vial for GC-MS analysis

Table S1. Method analytical and validation parameters for LC-SIM-MS analysis of carotenoids and tocopherols.

Compounds	Retention Time Precision		Working Range (μg/mL)	Limits		Correlation coefficient (R^2)	Area counts precision	
Compounds	Intra-day CV (%, n=6)	Inter-day CV (%, n = 6 x2)		LOQ (μg/mL)	LOD (μg/mL)		Intra-day CV (%, n = 6)	Inter-day CV (%, n = 6 x2)
(all-E)-violaxanthin	0.08	0.17	5-50	0.05	0.015	1.000	4.13	5.33
9-Z-neoxanthin	0.10	0.18	5-50	0.135	0.045	0.999	7.08	7.39
(all-E)-lactucaxanthin	0.07	0.16	5-50	0.36	0.12	1.000	4.67	5.41
(all-E)-lutein	0.06	0.15	5-50	0.075	0.025	1.000	2.49	4.96
(all-E)-zeaxanthin	0.10	0.15	5-50	0.135	0.045	1.000	5.17	7.10
(all-E)-β-carotene	0.08	0.16	5-50	0.39	0.13	1.000	4.95	6.09
α-tocopherol	0.21	0.32	10-100	1.57	0.52	0.999	8.91	9.25

CV, coefficient of variation; LOD, limits of detection; LOQ, limits of quantitation

Table S2. Method analytical and validation parameters for GC-MS analysis of sterols.

Sterols	Retention Time Precision		Working Range (µg/mL)	Limits		Correlation coefficient (R^2)	Area counts Precision	
	Intra-day CV (%, n=6)	Inter-day CV (%, n = 6 x2)		LOQ (µg/mL)	LOD (µg/mL)		Intra-day CV (%, n = 6)	Inter-day CV (%, n = 6 x2)
Campesterol	0.02	0.03	5-50	1.42	0.47	1.000	2.21	4.38
Stigmasterol	0.03	0.05	5-50	2.43	0.81	0.999	2.44	4.58
β-Sitosterol	0.03	0.04	5-50	2.90	0.97	0.998	2.34	6.16
α-Spinasterol	0.02	0.07	5-50	3.71	1.24	0.991	2.08	4.78

CV, coefficient of variation; LOD, limits of detection; LOQ, limits of quantitation

Figure **S3:** A characteristic mass fragmentation pattern of β-Sitosterol (trimethylsiloxy (TMS) derivative) observed in the present investigation.

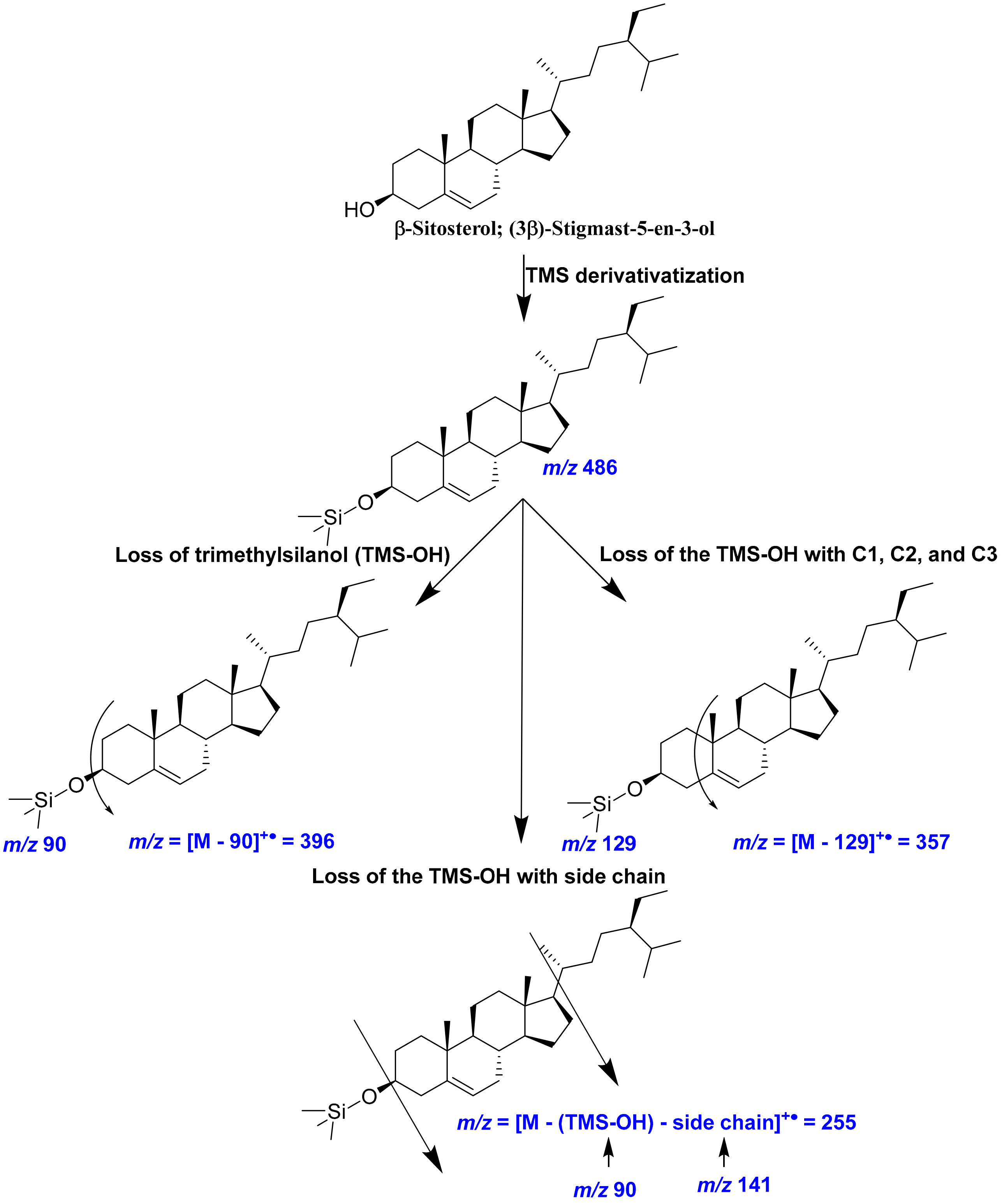

Figure S4: The characteristic GC-MS-fragmentation pattern of phytosterols identified in studied GLVs.

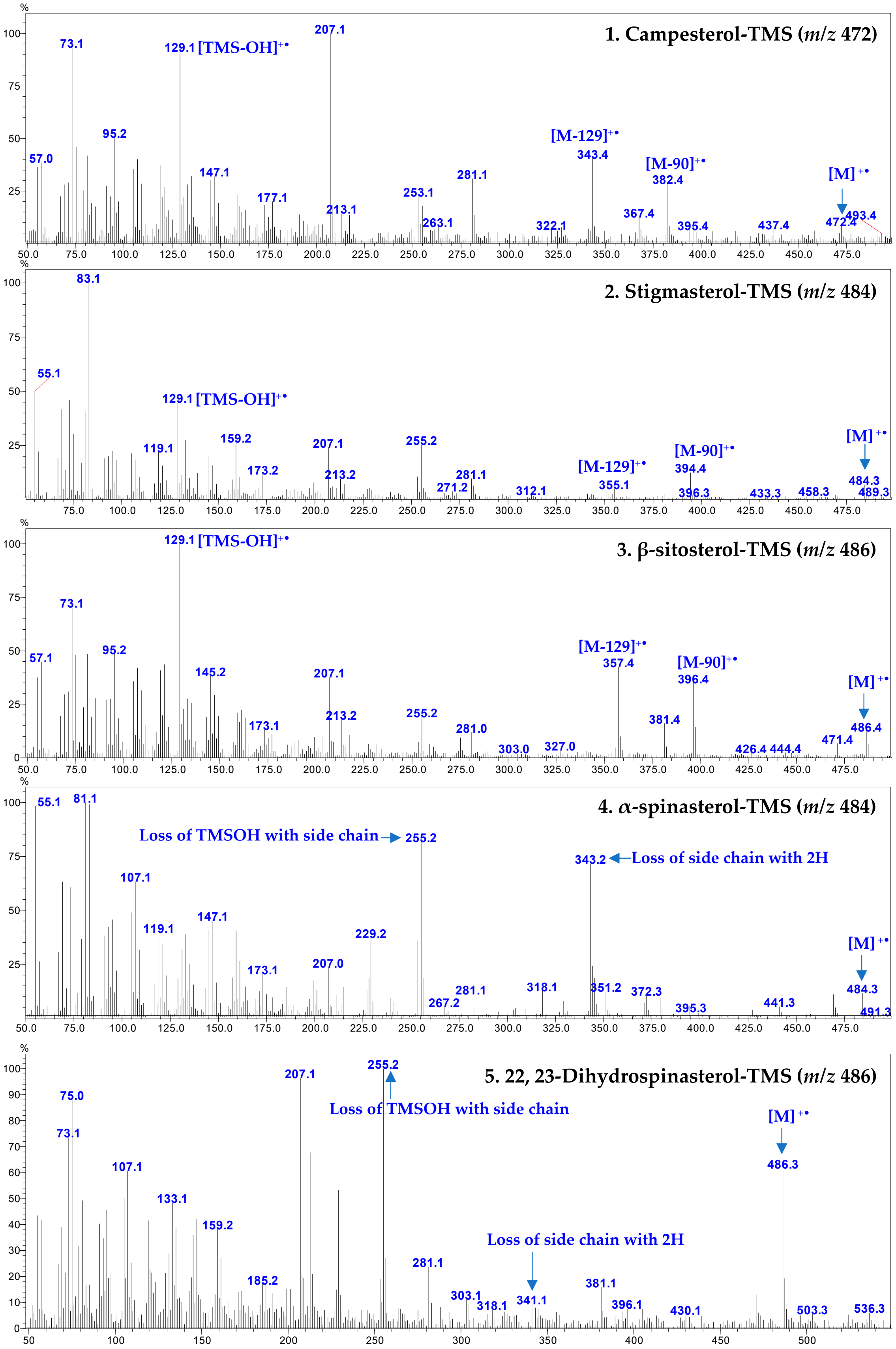

Supplement: Supplementary file 1 [file foods-11-03867-s001.zip › foods-2054291-supplementary.pdf]
